# Reserpine Causes Neuroendocrine Toxicity, Inducing Impairments in Cognition via Disturbing Hypothalamic–Pituitary–Thyroid Axis in Zebrafish

**DOI:** 10.3390/neurosci6020028

**Published:** 2025-04-01

**Authors:** Fengzhi Sun, Lijie Xia, Baokun Wang, Yanao Liu, Xiaotong Cui, Huijun Kang, Rostyslav Stoika, Kechun Liu, Meng Jin

**Affiliations:** 1 Biology Institute, Qilu University of Technology (Shandong Academy of Sciences), 28789 East Jingshi Road, Ji’nan 250103, China; sunhukong1999@163.com (F.S.);; 2 Engineering Research Center of Zebrafish Models for Human Diseases and Drug Screening of Shandong Province, 28789 East Jingshi Road, Ji’nan 250103, China; 3 Department of Regulation of Cell Proliferation and Apoptosis, Institute of Cell Biology, National Academy of Sciences of Ukraine, 16, Drahomanov Street 14, 79005 Lviv, Ukraine; stoika@cellbiol.lviv.ua

**Keywords:** reserpine, neuroendocrine, HPT axis, thyroid

## Abstract

Reserpine is used as a cheap and effective first-line antihypertensive, and presently, it is applied as treatment for difficult-to-control cases of hypertension. Despite its significance, reserpine’s neuroendocrine toxicity remains largely underexplored. Here, we investigated the effects of reserpine on development, locomotion, central nervous system (CNS) neurons, thyroid development, and the expression of genes related to neurodevelopment, endocrine, learning and memory, and depression in zebrafish exposed to different doses of reserpine ranging from 0.5 mg/L to 16 mg/L. The results of our study demonstrated that reserpine exerts dose-dependent toxicity on the neuroendocrine system (NES). An investigation into its underlying mechanism suggests that reserpine disrupted the hypothalamic–pituitary–thyroid (HPT) axis via down-regulating *hhex*, *tg*, and *tshβ* genes, impairing thyroid hormone synthesis and endocrine balance. Meanwhile, it affected neurodevelopment, as evidenced by the reduced expression of *gfap*, *gap43*, *mbp*, *syn2a*, and *tuba1b* genes, which compromised neuronal structure and function, while impaired neurotransmitter release and uptake could occur due to the suppression of *crhb* and *mao* genes. To conclude, these findings illustrate the interconnected impact of pathways involved in endocrine and neurodevelopment in reserpine-induced toxicity.

## 1. Introduction

Reserpine, an indole-derived compound, is isolated from the roots of *Rauwolfia serpentina*, a climbing shrub native to India. Approved by the FDA in 1955, it was one of the pioneering agents developed for the clinical management of hypertension [1]. Although reserpine is currently banned in some countries, it remains a useful treatment for some difficult-to-control cases of hypertension [2]. The side effects of reserpine in clinical use include depression and the development of Parkinson’s disease [3,4]. Administering a single subcutaneous dose of 5 mg/kg reserpine to neonatal rats on postnatal day 3 induces extensive neuronal degeneration across multiple brain regions, including the substantia nigra pars compacta, ventral tegmental area, striatum, hippocampus, locus coeruleus, amygdala, and cerebral cortex. In addition to toxic studies on rodents, it has been reported that acute reserpine treatment in zebrafish causes depression by inducing dopaminergic neuron damage in the brain [5]. In another study, reserpine induced Leydig cell hyperplasia in rabbits, which can consequently alter testosterone synthesis [6]. A reduction in serum testosterone levels and epididymal weight can be observed. This suggests that reserpine has adverse effects on the development and hormone secretion of the reproductive organs. Additionally, reserpine produced typical dyskinesia [7] and nociceptive sensitization [8]. However, it remains largely unknown whether reserpine affects the NES.

In vertebrates, the NES is characterized by cells that are organized into both distinct organs and diffuse structures, all of which collectively co-produce amine and peptide hormones/neurotransmitters, along with specific markers of neural differentiation [9]. The NES is distributed throughout the body, with the gastrointestinal tract and pancreas exhibiting about 17 different neuroendocrine cell types [10]. The hypothalamic–pituitary (HP) axis is integral to the NES and crucial for regulating physiological and behavioral homeostasis, as well as for coordinating essential bodily functions [11]. The HP axis can be divided downward into the HPT axis [12], hypothalamic–pituitary–adrenal (HPA) axis [13], and hypothalamic–pituitary–gonadal (HPG) axis [14]. The HPT axis is a classic example of the NES that regulates different functions of organisms during development. Thyroid hormones (THs), including tri-iodothyronine (T3) and thyroxine (T4), secreted by the thyroid gland as a target organ of the HPT axis, are important for the process and maintenance of normal physiological development, especially in the CNS. In addition, brain maturation during pregnancy is influenced by the involvement of THs [15,16]. Congenital TH deficiency can cause devastating neurocognitive outcomes, such as deficits in memory and attention [17]. Congenital hypothyroidism may also cause significant intellectual disability [18]. Except for the effect on CNS, *D3KO* mice born with hypothyroidism are observed to have impaired growth and fertility [19]. Delayed bone formation and mineralization are observed in juveniles with hypothyroidism [20]. Thus, the investigation of reserpine’s impact on neuroendocrine and cognitive function is of significant importance.

Zebrafish have been used since the 1970s in scientific research as a typical vertebrate model organism [21], with a high degree of similarity to the human genome and complete NES. The nervous system of zebrafish is analogous to that in humans, incorporating the hypothalamus and spinal cord. There is also a great deal of consistency in the endocrine organs, such as the pituitary gland, thyroid gland, adrenal glands, and gonads. Previous research indicates that drugs can induce neuroendocrine toxicity, including meclizine cortisone and donepezil [22,23,24].

In order to further investigate the toxicology of reserpine and clarify the mechanism of its side effects, the present study investigated the dose-dependent effects of reserpine-induced neuroendocrine toxicity using zebrafish as an experimental model. We examined the toxic effects of reserpine on development, the NES, and transcript levels of genes associated with neurodevelopment, endocrinology, and cognition. To complete the above study, we performed zebrafish live imaging, multi-behavior assays, phenotype scoring, quantitative real-time PCR, and other investigations.

## 2. Materials and Methods

### 2.1. Animals and Reagents

The wild-type zebrafish line AB, the transgenic zebrafish (*elavl3: EGFP*), and the transgenic zebrafish (*tg: EGFP*) were used in this study. Two transgenic zebrafish strains were labeled with green fluorescence in neurons of the CNS and thyroid gland, respectively [25]. Zebrafish were sourced from the China Zebrafish Resource Center and maintained in compliance with the NIH Guide for the Care and Use of Laboratory Animals (No. 8023). Methylene blue, 1-phenyl-2-thiourea (PTU), and tricaine (MKCJ6340) were acquired from Sigma (St. Louis, MO, USA). Dimethyl sulfoxide (DMSO) was purchased from Solarbio (Beijing, China). Methylcellulose and reserpine were bought from Aladdin (Shanghai, China). All the chemicals and reagents utilized in the present study were analytical grade.

### 2.2. Zebrafish Maintenance and Chemical Exposure

Adult zebrafish from three different strains were housed at 28 °C ± 0.5 °C under a 14/10 h light/dark cycle, with live brine shrimp administered as feed twice daily. After natural mating, the embryos were collected and subsequently incubated in a bathing medium containing 2 μg/mL methylene blue, which served as a disinfectant [26]. In total, 0.003% PTU, acting as melanogenesis inhibitors [27], was added to the bathing medium. Moreover, 4 hpf (hours post fertilization) embryos were used for the experiments to be examined under a dissecting microscope (Carl Zeiss, Jena, Germany). Embryos that progressed normally to the blastula stage were chosen for subsequent analyses.

The selected embryos (4 hpf) were distributed into six-well plates at random and exposed to different levels of reserpine dissolved in the bathing medium, respectively [28]. These embryos were exposed to a suitable temperature, 28 °C ± 0.5 °C [29]. Reserpine was dissolved in 0.1% (*v*/*v*) of DMSO and the solution was agitated until no undissolved particles were visible. The various concentrations of reserpine were prepared through serial dilution in standard bathing solution. The drugs were added to the embryos from 4 hpf to 144 hpf once a day, ranging from 1/5 of LC1 to LC50 (0.5 mg/L, 1 mg/L, and 2 mg/L). The zebrafish in the control group were treated with 0.1% (*v*/*v*) of DMSO. Any dead embryos were discarded after every dosing. For each vehicle control group and exposure group, three replicates (*n* = 3) were conducted.

### 2.3. Developmental Toxicity Evaluation

The development of zebrafish from 24 to 144 hpf was observed and photographed using a Zeiss microscope (Jena, Germany). Mortality, the hatching rate, and the malformation rate were considered indicators for the evaluation of developmental toxicity. From 24 hpf to 144 hpf, mortality was assessed. At 48 hpf and 72 hpf, the hatching rate was documented, and at 144 hpf, the malformation rate was examined. Mortality was demonstrated in the failure of somite development, larval coagulation, a missing heartbeat, and a nondetached tail. The malformation rate was evaluated according to the following indicators: bent spinal (BS), pericardial edema (PE), the absence of a swimming bladder (AB), tail malformation (TM), yolk sac malformation (YM), and small eyes (SE). Morphology changes in the embryos were recorded.

### 2.4. Neurotoxicity Evaluation

After 6 days dosing, regarding the transgenic zebrafish (144 hpf), 8 individuals from the vehicle control and exposure groups were randomly selected and anesthetized for visual observation and image acquisition. The development of the CNS neurons’ formation was obtained by visualizing micrographs in a fluorescence stereomicroscope (Carl Zeiss, Jena, Germany). We performed statistical analyses of dopamine neuronal region lengths in the different doses of the reserpine-exposed group compared to the control group. The integrated optical density (IOD) of the head and notochord, marked by yellow arrows, was then measured using Image Pro software 6.0 (Media Cybernetics, Bethesda, MD, USA). To assess the differentiation of CNS neurons following reserpine treatment, a statistical analysis of the fluorescence intensity in differentiated CNS neurons was performed, comparing the reserpine-treated groups to the control group. Each group underwent three replicates.

### 2.5. Locomotion Recording—From This Point, My Changes Are Denoted in Blue

The locomotor behaviors of *AB* strain zebrafish were monitored over a 144 h period following reserpine exposure. To minimize the influence of physical abnormalities on behavioral outcomes, only larvae displaying no visible deformities were included in the movement analysis. Three behavioral tests were performed, general locomotion, light/dark transition, and thigmotaxis assessment, to evaluate reserpine’s effects on zebrafish motor function. Larvae were harvested and rinsed in a bathing solution after six days of reserpine treatment across the experimental groups. The zebrafish larvae from every group were put in 48-well plates (one at a time and containing 1 larva per well) to monitor their locomotor activity after 144 hpf exposure to reserpine at 28 ± 0.5 °C. For general locomotion, the light was off. A light and dark challenge based on the general behavior test was added to the periodic change in light to zebrafish. We monitored changes in locomotion during lighting [30]. The wild-type zebrafish larvae of line AB were placed in 48-well plates. The light and dark challenge was conducted in 60 min. It was made up of 3 cycles of light/dark phases (with 20 min of illumination for each phase). Each larva was recorded for 20 min with an automated computerized video-tracking system (Viewpoint, Lyon, France) after timing 10 min for acclimation. The digital tracks of fish movements were analyzed and the average speed per minute was calculated by using the Zeblab software 3.1 (Viewpoint, Lyon, France). Thigmotaxis is commonly utilized as an indicator of anxiety-like behavior, and it is increasingly being applied in zebrafish models [31]. Male and female adult zebrafish of the AB wild type were subject to experiments in this test. To obtain the accurate tracking of the swimming behavior of individual fish, we first adjusted the settings of the automated video-recording system before the beginning of the experiment: acclimatization (minutes 10) and visual motor challenge (minutes 10). Next, the zebrafish after cleaning were transferred into a 12-well plate. A circular area covering 50% of the well area was delineated in each well of the 12-well plate. To assess thigmotaxis in zebrafish larvae, two metrics were established: the outer zone distance ratio and outer zone duration ratio. The outer zone distance ratio was calculated by dividing the distance moved in the peripheral region by the total distance and multiplying by 100. Correspondingly, the central zone duration ratio was determined through the same calculation method using time measurements. Six-day post-fertilization larvae freely swam in individual wells, with their movements recorded using a video-tracking system (Viewpoint, Lyon, France).

### 2.6. Assessment of Thyroid Development

Following 6 days of reserpine exposure, zebrafish at 144 hpf were anesthetized, after which 12 individuals from each group were randomly selected for visual observation and image acquisition. Each group underwent three replicates. Transgenic zebrafish (tg: EGFP) were utilized to evaluate thyroid gland development. Photographic images were captured using a confocal scanning microscope (Zeiss, Jena, Germany). Endocrine toxicity was identified by the green fluorescent labeled area. Twelve zebrafish larvae were employed in each group.

### 2.7. Real-Time Quantitative PCR (qPCR)

The zebrafish larvae (144 hpf) were collected and used for RNA extraction. Total RNA was extracted from 30 individuals with the help of EASY spin Plus RNA Mini Kit. (312423AX; Aidlab Biotechnologies; Beijing, China) in accordance with the manufacturer’s instructions. Products of reverse transcription, cDNA, were carried out using the PrimeScript™ RT Master Mix (Takara, Tokyo, Japan). qPCR was performed using SYBR^®^ Premix DimerEraser™ (Takara, Tokyo, Japan) and the Light Cycler^®^ 96 System (Roche, Basel, Switzerland). Reverse transcription and real-time quantitative PCR (qPCR) were carried out in accordance with the manufacturer’s instructions. Runs were carried out in triplicate using the housekeeping gene *rpl13a* to normalize the mRNA level of target genes. The primers used are listed in Table 1.

### 2.8. Statistical Analysis

The results were analyzed by using one-way ANOVA followed by Dunnett’s post hoc test with Graph Pad Prism 7.0 (GraphPad Software). The results were presented as mean ± SEM. *p* < 0.05 was considered significant.

## 3. Results

### 3.1. Reserpine Induces Dose-Dependent Developmental Toxicity

**Figure 1:**
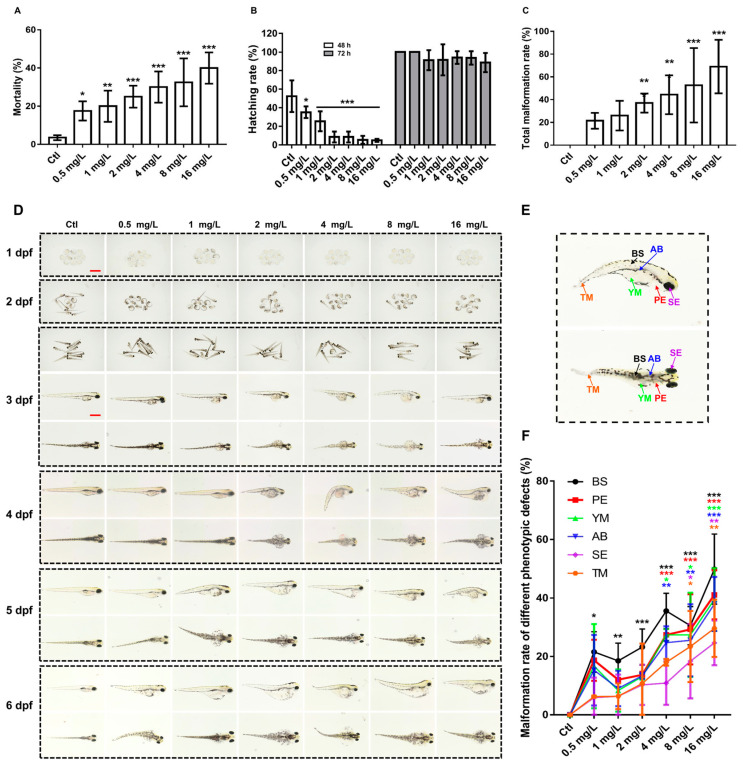
Reserpine-induced developmental impacts on zebrafish larvae. (**A**) Reserpine-induced mortality at 144 hpf. (**B**) Embryonic hatching rates at 48 and 72 hpf under varying concentrations of reserpine. (**C**) Cumulative malformation incidence. (**D**) Morphological progression from 1 to 6 dpf across treatment groups. (**E**) Representative developmental abnormality. (**F**) The percentage of different malformation. *n* = 12 per group. Scale bar, 1000 μm. * *p* < 0.05, ** *p* < 0.01, *** *p* < 0.001 vs. Ctl.

Reserpine exposure in zebrafish over 144 h yielded LC1 and LC50 values of 4 mg/L and 16 mg/L, respectively. Based on these toxicity thresholds, we evaluated dose-dependent effects using sub-LC1 concentrations (0.5, 1, and 2 mg/L), LC1, LC50, and an intermediate concentration between LC1 and LC50. Figure 1A demonstrates concentration-dependent mortality, with significant increases observed above 2 mg/L. Embryonic development analysis revealed delayed hatching at 48 hpf, though normal hatching rates were observed by 72 hpf (Figure 1B). Developmental abnormalities showed dose-dependent escalation from 2 mg/L onward (Figure 1C), presenting multiple morphological defects: bent spinal (BS), pericardial edema (PE), the absence of a swimming bladder (AB), tail malformation (TM), yolk sac malformation (YM), and small eyes (SE). Zebrafish had the highest malformation rate for all phenotypic defects when exposed to 16 mg/L of reserpine. There was a dose-dependent increment in the proportion of each malformation (Figure 1F).

### 3.2. Effects of Reserpine on CNS Neuron Differentiation

**Figure 2:**
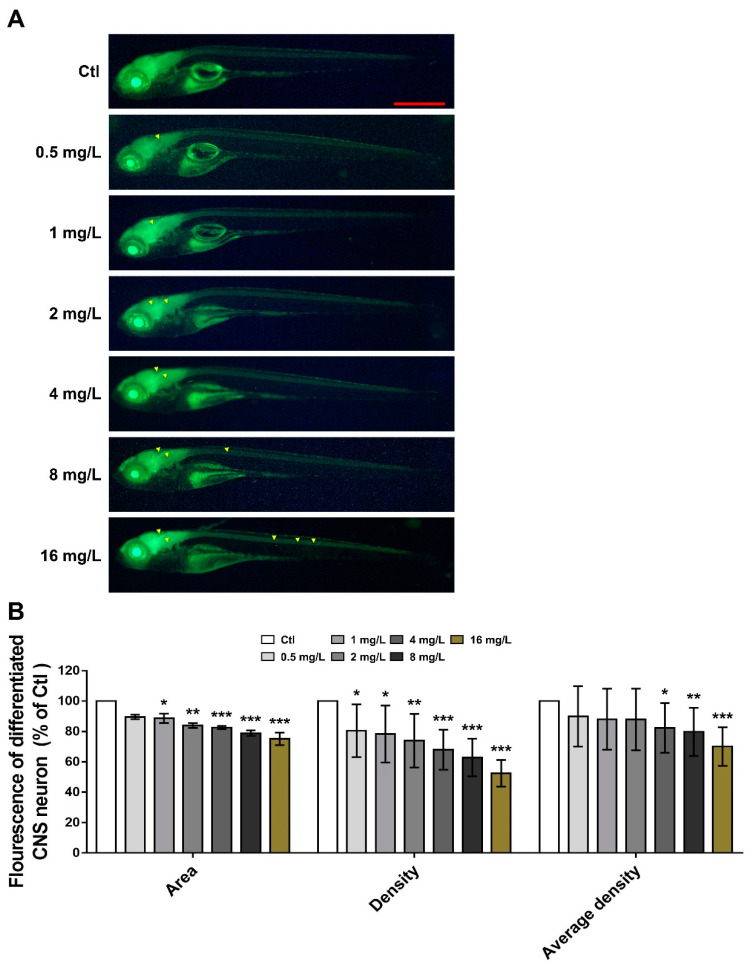
CNS neuron development in larval zebrafish treated with reserpine. (**A**) Representative illustrations of central neuronal differentiation in *elval3: GFP* zebrafish subjected to reserpine therapy; the manifestation of CNS differentiation is denoted by yellow arrows. (**B**) Statistical analysis of the CNS differentiation in the reserpine group and the control group; the CNS differentiation is indicated by fluorescence intensity. *n* = 12 per group. Scale bar, 100 μm. * *p* < 0.05, ** *p* < 0.01, *** *p* < 0.001 vs. Ctl.

To analyze the impacts of reserpine on CNS neuron differentiation, we quantified green fluorescence in area, density, and average density by using *Tg (elavl3: EGFP),* at which CNS cells were fluorescently labeled. We found that reserpine could inhibit CNS neuronal differentiation in a dose-dependent manner (Figure 2). The effect of reserpine on zebrafish brain neurodevelopment was greater than that on spinal pyramidal neurons, with significant reductions in both brain fluorescence intensity and spinal fluorescence intensity at concentrations greater than 2 mg/L.

### 3.3. Dose-Dependent Effects of Reserpine on Thyroid Development

**Figure 3:**
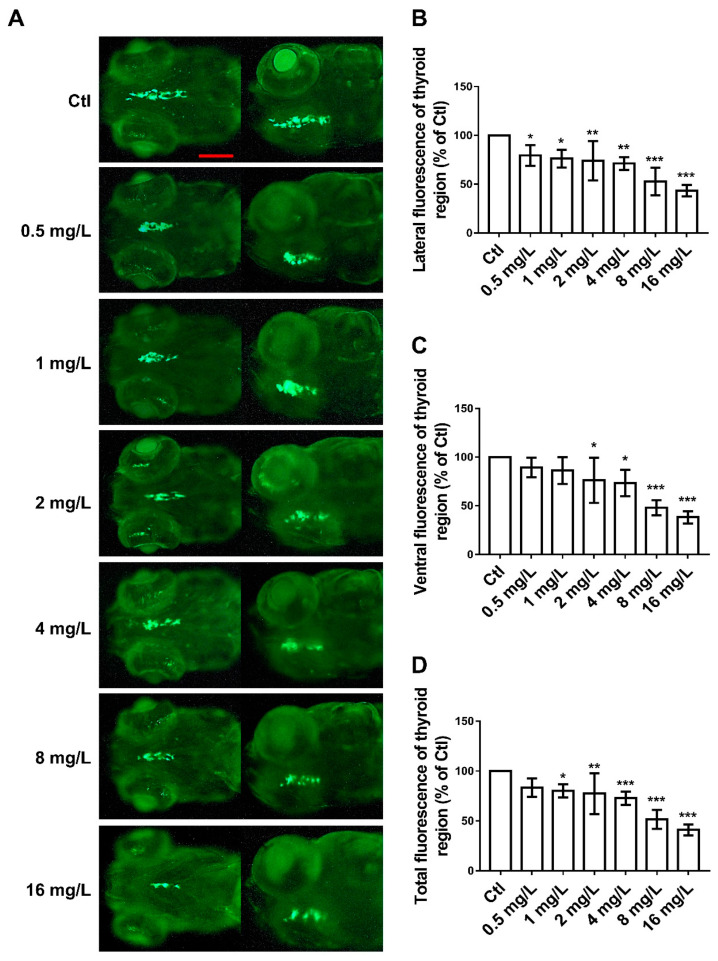
Dose-dependent effects of reserpine on thyroid development. (**A**) Representative images of thyroid development in zebrafish, where development of thyroid is indicated by fluorescence. (**B**) Lateral fluorescence of zebrafish thyroid region at 72 hpf. (**C**) Ventral fluorescence of zebrafish thyroid region at 72 hpf. (**D**) Total fluorescence of zebrafish thyroid region at 72 hpf. * *p* < 0.05, ** *p* < 0.01, *** *p* < 0.001 vs. Ctl.

To investigate the effects of reserpine on zebrafish endocrine function, we examined thyroid gland development in zebrafish following reserpine treatment at 144 hpf. We employed *Tg* (*tg: EGFP*) zebrafish, whose thyroid follicular cells were fluorescently labeled, as our experimental model. The analysis was conducted through live imaging using a confocal microscope. As shown in Figure 3, we observed and recorded the lateral and ventral fluorescence area of zebrafish. The fluorescence area was significantly reduced when the concentration of reserpine was greater than 8 mg/L (Figure 3A–C). For the total fluorescence of thyroid, the area was significantly reduced when the concentration of reserpine was greater than 4 mg/L (Figure 3D). Reserpine had a dose-dependent toxic effect on thyroid development based the trend of decreasing fluorescence area.

### 3.4. Reserpine Injures Locomotor Capacity of Zebrafish in a Dose-Dependent Manner

**Figure 4:**
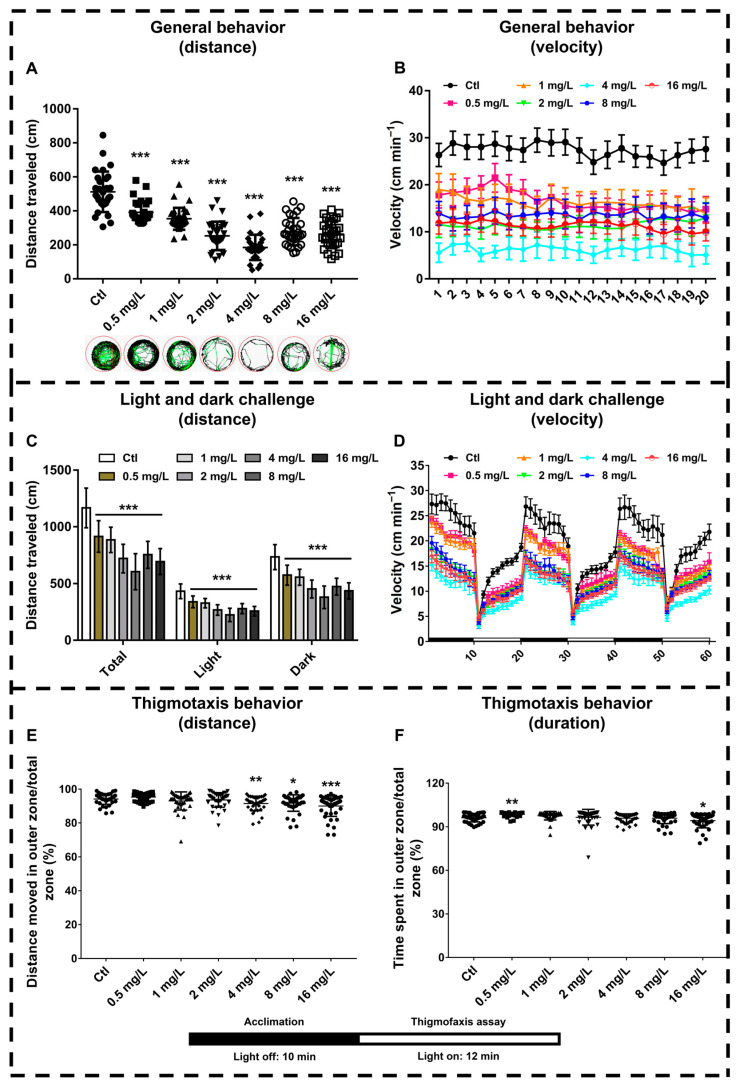
The locomotor behavior of zebrafish larvae following exposure to reserpine. (**A**) Locomotor activity patterns across the experimental groups, illustrated through digital motion traces. Movement trajectories are color-coded: black for low velocity, green for moderate velocity, and red for high velocity. (**B**) Average swimming speed for general locomotion. (**C**) Swimming distance for light and dark challenge; the result is expressed as Total, Dark and Light. (**D**) Distance traveled in light/dark challenge exposed to different doses of reserpine at 72 hpf. (**E**) The percentage of the moving distance of zebrafish in the peripheral area to the total area. (**F**) The percentage of the duration that zebrafish larvae spend in the total area compared to the peripheral area. * *p* < 0.05, ** *p* < 0.01, *** *p* < 0.001 vs. Ctl.

Taking into account the relationship between behavior and neuroendocrine status, we conducted three different locomotor assays including general locomotion, light/dark challenge, and thigmotaxis locomotion. For general locomotion, we recorded the movement trajectory, distance, and speed of zebrafish after reserpine treatment. In Figure 4A,B, the movement distance and speed of zebrafish larvae show a significant downward trend under the influence of high-concentration reserpine. The changes in movement were observed in the digital tracks shown. For the light/dark challenge, the reserpine-treated group showed a significant decrease in the distance traveled compared to the control group (Figure 4C). We also found that control zebrafish showed a sharp decrease in movement speed when the light was on, then a gradual increase and a sharp increase in movement speed when the light was off, and then a gradual decrease, which was a more moderate trend in the reserpine-treated group (Figure 4D). When zebrafish are placed in a well plate, the zebrafish show a tendency to move closer to the walls of the well, and depressive status decreases this tendency behavior. For the thigmotaxis assay, the reserpine-treated group exhibited depression-like behavior compared to the control. Reserpine at a relatively high dose significantly reduced the distance traveled by the zebrafish to swim in the outer circle to touch the well wall, as well as the time spent swimming in the outer circle (Figure 4E,F).

### 3.5. The Effects of Reserpine on Transcript Levels of NES- and Cognition-Related Genes

**Figure 5:**
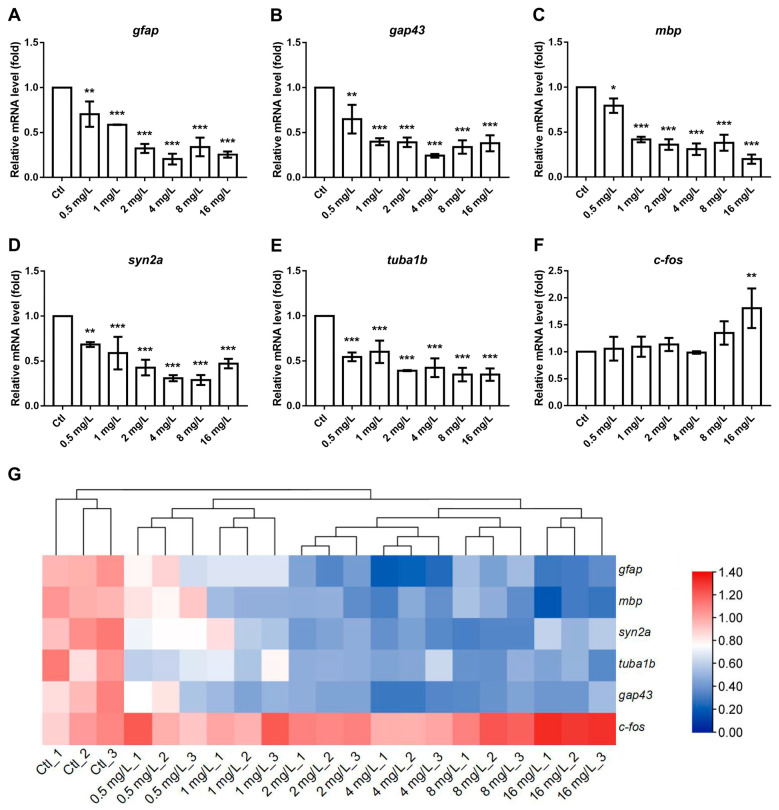
Effect of reserpine on transcript levels of neurodevelopment-related genes. (**A**–**F**) Transcription levels of genes involved in neurodevelopment, including glial fibrillary acidic protein (*gfap*), growth associated protein 43 (*gap43*), myelin basic protein (*mbp*), synapsin 2a (*syn2a*), tubulin alpha 1b (*tuba1b*), and cellular oncogene fos (*c-fos*). (**G**) Colors indicate the direction and intensity of changes in gene transcript levels after 144 h of reserpine treatment. * *p* < 0.05, ** *p* < 0.01, *** *p* < 0.001 vs. Ctl.

**Figure 6:**
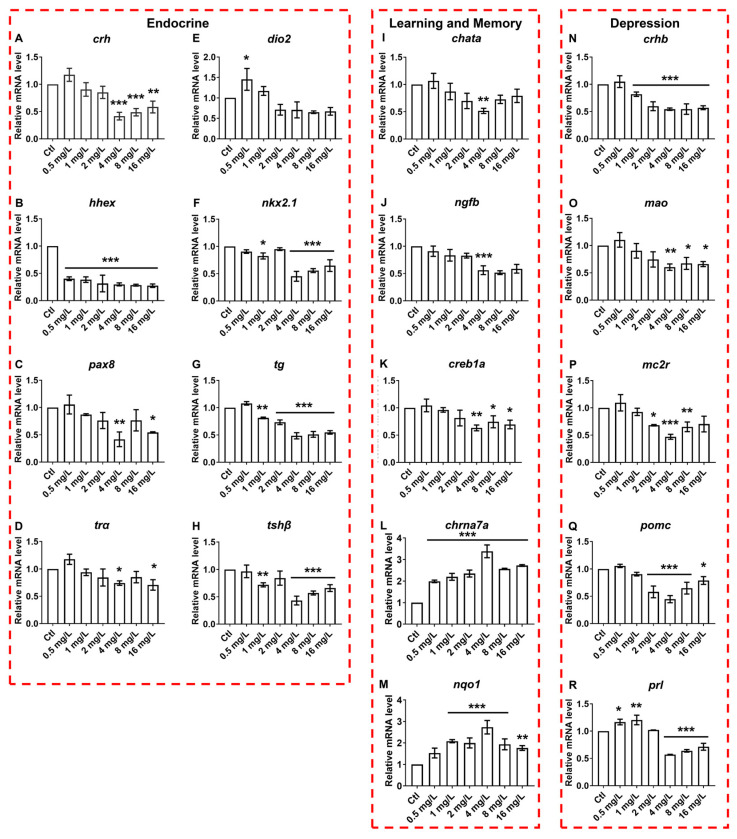
Effect of reserpine on transcript levels of endocrine and cognition-related genes. (**A**–**H**) Transcription levels of endocrine-related genes, including corticotropin-releasing hormone (*crh*), hematopoietically expressed homeobox (*hhex*), paired box 8 (*pax8*), t cell receptor alpha locus (*tra*), deiodinases type Il (*dio2*), thyroid transcription factor 1 (*nkx2.1*), thyroglobulin (*tg*), thyroid-stimulating hormone subunit β (*tshβ*). (**I**–**M**) Transcription levels of genes involved in learning memory including choline acetyltransferase a (*chata*), nerve growth-factor beta (*ngfb*), Cyclic AMP-responsive element-binding protein 1 a (*creb1a*), Cholinergic Receptor Nicotinic Alpha 7 Subunit a (*chrna7a*), quinone oxidoreductase 1 (*nqo1*). (**N**–**R**) Transcript levels of genes associated with depression including corticotropin-releasing hormone binding (*crhb*), monoamine oxidases (*mao*), melanocortin 2 receptor (*mc2r*), proopiomelanocortin (*pomc*), prolactin (*prl*). * *p* < 0.05, ** *p* < 0.01, *** *p* < 0.001 vs. Ctl.

To investigate the effects of reserpine on mechanisms of neuroendocrine regulation, we assessed the transcript levels of genes associated with neurodevelopment, endocrinology and cognition. Regarding the genes implicated in neural development, a dose-dependent increase in the transcript levels of *c-fos* in zebrafish was identified. In contrast, a dose-dependent decrease in the transcript levels of *gfap, gap43, mbp, syn2a,* and *tuba1b* were detected (Figure 5A–E). In the case of genes associated with endocrine processes, the transcript levels of *hhex* were significantly reduced in all reserpine-treated groups (Figure 6B). Moreover, compared to the control group, the transcript levels of genes such as *crh, nkx2.1*, *pax8*, *tg*, *tra*, and *tshβ* all exhibited a downward trend (Figure 6A,C,F–H). Learning, memory and depression are essential indicators of cognitive ability. In related gene transcript levels assays, *chrna7a* and *nqo1* showed significant increases at almost all concentrations compared to the controls, and there was a significant decrease in the transcript levels of *crhb* when the concentration was greater than 2 mg/L, while *chata*, *ngfb*, *creb1a*, *mao*, *mc2r*, and *pomc* were most prominent at a treatment concentration of 4 mg/L (Figure 6I–Q). Interestingly, after low concentrations of reserpine treatment, the transcript levels of *prl* were elevated, but when the concentration of reserpine was increased, the transcript levels of *prl* were instead reduced (Figure 6R).

## 4. Discussion

Antihypertensive drugs recommended by the World Health Organization are divided into three categories, including thiazides, angiotensin, and calcium channel blockers [32]. Reserpine, as one of the first antihypertensive drugs, is not the first choice for antihypertensive drugs because of its side effects. However, reserpine has a unique effect on some kinds of refractory hypertension; thus, we still have to pay attention to its negative effects on organisms. Here, we found that reserpine affected thyroid function and neurodevelopment by impairing the HPT axis, which led to endocrine disruption with effects on neurotransmitter release and uptake causing neuroendocrine toxicity and contributing to neurological disorders such as injured cognition and depression.

### 4.1. The Dose-Dependently Developmental Toxicity of Reserpine

In order to investigate the developmental toxicity of reserpine exposure, we analyzed its effects on zebrafish at different concentration levels ranging from 0.5 mg/L to 16 mg/L. We found dose-dependent adverse effects of reserpine exposure on mortality, hatchability, malformations and the incidence of different malformations in zebrafish. Previous studies have shown that the reserpine treatment of pregnant rats resulted in an increased stillbirth rate and decreased weight loss and survival of pups [33], and high-dose reserpine exposure led to higher rates of malformations in pups [34]. A study has reported that a woman exposed to reserpine (0.5 mg/day) during the first six weeks of pregnancy; the fetus was born with severe malformations, and on examination, it was chromosomally normal, which can be ruled out as being due to a chromosomal deletion [35]. The above studies are to some extent consistent with our experimental results. The ability of reserpine to cause developmental toxicity has been demonstrated.

### 4.2. Reserpine Induced Neurotoxic Characteristics

Reserpine exposure led to abnormal CNS neuron differentiation. In our experiments, zebrafish exhibited a significant impairment in CNS neurons after exposure to a concentration of 4 mg/L reserpine. Similarly, in a previous study of rifampicin toxicity to the rat CNS, exposure to reserpine resulted in significant neuronal damage in the hippocampus, substantia nigra, and striatum of the brain in rat pups [36]. Distance travel data showed that 2 mg/L and 4 mg/L of reserpine exhibited a reduced travel distance in both general and light and dark challenge distances. This phenomenon may be due to the self-protective mechanisms underlying neurotoxic effects triggered by varying concentrations of reserpine [37]. When zebrafish were treated with 2 mg/L and 4 mg/L of reserpine, a significant reduction in travel distance was due to nervous system damage. As the reserpine concentration increased to 8 mg/L and 16 mg/L, cellular protective mechanisms might have been activated, resulting in the partial recovery of the nervous system [38]. Despite this recovery, the decrease in the travel distance of zebrafish remained statistically significant compared to the control (*** *p* < 0.001). In addition, the light/dark challenge is a valuable indicator for detecting the neurodevelopmental status of zebrafish. In a previous study, polybrominated diphenyl ether exposure caused neurological damage in zebrafish, and during the light/dark transition, zebrafish showed a decrease in light sensitivity, with a smooth trend in speed change compared to the control group [39], which is consistent with our results.

To explore the influence of reserpine on the CNS of zebrafish at the molecular level, a further analysis of related genes was conducted. The SYN2A, a biomarker of synapse formation, is a neuronal phosphoprotein and is involved in regulating neurotransmitter release and synaptogenesis [40]. In a previous study, it was reported that diphenyl phosphate treatment induced neurotoxicity and decreased *syn2a* expression in zebrafish. This is consistent with our experimental results that reserpine might cause neurotoxicity by inhibiting synapse formation. *tuba1b* encodes a protein that is a key component of microtubules, which are essential for the construction of brain structures. The proper expression of *tuba1b* is crucial for this process. Previous studies have shown that the transcriptional level of *tuba1b* was reduced in zebrafish with abnormalities in the HPT axis [41]. In our study, after treatment with reserpine at all concentrations, the transcriptional level of *tuba1b* was significantly down-regulated. This implied that reserpine exposure slowed down the development of the nervous system in zebrafish. The encoded protein of *gap43* is mainly found in the presynaptic membrane and is essential for promoting neuronal growth as well as synaptogenesis [42]. The abnormal expression of *gap43* is associated with various nervous system diseases. When nerves are damaged, the expression of this gene is upregulated to defend this injury. On the other hand, during development, the insufficient expression of *gap43* can impair synapse formation, leading to abnormal neural development [43]. Previous studies have shown that olaquindox induces neurodevelopmental toxicity in zebrafish through the down-regulation of *gap43* expression [44]. This is consistent with the results of our experiment. Similarly, *gfap*, a key regulatory gene for astrocytes that is usually upregulated when nerves are damaged and need to be repaired [45], showed a reduced transcript level in our experiments. The generation of axonal myelin is a critical step in the development of the zebrafish CNS, and the *mbp* counterpart protein is a marker for myelin generation [46,47]. In the present experiment, after treatment with low doses of reserpine, the transcriptional level of *mbp* in zebrafish decreased significantly. Reserpine may have affected the formation of myelin sheaths and caused nerve conduction to proceed normally. *c-fos* is an important marker gene in neuronal activity [48]. In our investigation, a significant upregulation in expression levels was exclusively observed following exposure to elevated reserpine concentrations. This phenomenon may be attributed to the time interval between the collection of zebrafish larvae and the administration of the drug. As an immediate-early gene, the expression level of *c-fos* recovered after treatment with low doses of reserpine. In a previous study, *c-fos* expression in rats subjected to electroconvulsive stimulation lasted for only one hour [49].

### 4.3. The Effect of Reserpine Exposure on the Endocrine System

The thyroid gland is an important endocrine organ of living beings and one of the important target organs of the neuroendocrine system. The study of the thyrotoxicity of reserpine is of great significance in analyzing its effects on the endocrine system. It has been reported that TH secretion decreased in chickens after reserpine exposure [50]. In addition, researchers measured the amount of organic radioactive iodine (125-iodine + 125-iodide) in the feces and glands of rats treated with reserpine for two weeks. The results showed that reserpine exposure led to a decline in thyroid function [51], suggesting that the rats treated with reserpine for 14 days were in a state of hypothyroidism. In our experiments, by analyzing the zebrafish thyroid region, we similarly found that reserpine exposure caused damage to the thyroid gland.

In addition, we analyzed the transcript levels of endocrine-related genes. The expression levels of *crh*, *dio2*, *pax8*, and *tra* were increased after exposure to lower concentrations of reserpine, but this was not significant. This may be due to the hormetic effects of reserpine on the transcript levels of endocrine-related genes [52]. When we elevated the exposure concentration of reserpine, the transcript levels of endocrine-related genes were reduced to varying degrees. This suggested that reserpine negatively affected the endocrine system.

### 4.4. Mechanisms of Reserpine-Induced Neuroendocrine Toxicity That Lead to Cognitive Impairments

The NES includes the hypothalamus, pituitary gland, and end target organs or target tissues, which function together as an axis. In our study, the thyroid gland, a target organ of the HPT axis, showed a decrease in its size after reserpine exposure. TH deficiency led to the abnormal development of almost all organs [53,54] and, of course, other neuroendocrine target organs. At the same time, the development of the nervous system is inhibited. The thyroid gland, CNS, and other neuroendocrine target organs interact with each other to form feedback regulation, which ultimately leads to neuroendocrine toxicity.

Cognitive impairments include depression, schizophrenia, anxiety, and others [55]. All of these disorders have a strong link to the neuroendocrine system. The development of these disorders often leads to a decline in learning and memory skills. We analyzed the transcript levels of genes associated with depression, including *crhb*, *mao*, *mc2r*, *pomc*, and *prl*, as well as genes related to learning and memory, including *chata*, *creb1a*, *ngfb*, *chrna7a*, and *nqo1*. *crhb* is the gene for the corticotropin-releasing hormone-binding protein, which binds to the corticotropin-releasing hormone and regulates the stabilization of the neuroendocrine system [56]. *mao* encodes a protein that catalyzes the oxidative deamination of monoamine neurotransmitters, such as norepinephrine, dopamine, and serotonin, thereby regulating the levels of these neurotransmitters [57]. Mutations in *mao* can cause major depression. *mc2r* is normally expressed to levels necessary for the normal synthesis and secretion of adrenocorticotropic steroid hormones [58]. *pomc* is implicated in the synthesis of a variety of biologically active peptides, among which adrenocorticotropic hormone improves the body’s ability to resist stress [59]. *prl*-encoded prolactin is involved in the development of the major gonads. In addition, it has a regulatory effect on insulin secretion [60]. In our experiments, the down-regulation of transcript levels of the above mentioned genes by high concentrations of reserpine exposure suggested, in part, that reserpine might contribute to injured cognitive behaviors by affecting neuroendocrine functions. *chata* is responsible for regulating the synthesis of the neurotransmitter acetylcholine, which is essential for nerve signaling in the neuromuscular junction. *creb1a* regulates the expression of genes related to learning and memory to help consolidate memory [61]. *ngfb* is responsible for encoding proteins that can stimulate the growth and branching of neuronal axons [62]. *chrna7a* expression enhances neuronal signaling that leads to cognitive enhancement [63]. In a previous study, it was found that when BPA induced learning memory deficits in mice, the transcript level of *nqo1* was significantly reduced [64]. Here, the transcription levels of the above genes related to learning and memory were abnormal, indicating that reserpine may cause a decline in the learning and memory ability of zebrafish by inducing neuroendocrine toxicity, and this correlates with the results of thigmotaxis locomotion.

## 5. Conclusions

Our findings revealed that reserpine induced dose-dependent neuroendocrine toxicity in zebrafish, leading to cognitive decline and disruptions in neurotransmitter and hormone secretion. This neuroendocrine dysregulation was accompanied by neurotransmitter imbalances, highlighting the interconnected impact of these pathways in multifaceted toxicological toxicity effects of reserpine. By impairing the HPT axis and neurodevelopment, reserpine exposure causes long-term neuropsychiatric and metabolic disturbances. These insights highlight the need for an increased awareness of reserpine’s potential to induce NES dysfunction and for the vigilant monitoring of its neuroendocrine toxicity.

## Figures and Tables

**Table 1:** Primers used for quantitative real-time PCR reactions.

Gene Symbol	Forward Primer Sequence (5'→3')	Reverse Primer Sequence (5'→3')
*gfap*	5'-GGATGCAGCCAATCGTAAT	5'-TTCCAGGTCACAGGTCAG
*gap43*	5'-CAGCCGACGTGCCTGAA	5'-GGATTCCTCAGCAGCGTCTG
*mbp*	AATCAGCAGGTTCTTCGGAGGAGA	AAGAAATGCACGACAGGGTTGACG
*syn2a*	GTGACCATGCCAGCATTTC	TGGTTCTCCACTTTCACCTT
*tuba1b*	AATCACCAATGCTTGCTTCGAGCC	TTCACGTCTTTGGGTACCACG
*c-fos*	GCTCCTGGCTAAAGCGGAGCTG	GACGTGTAGGTGGTGCAGGCTGG
*crh*	ATCTCAAGGAAGGCGAATAGA	AACATCGATGGAAAGTGATGA
*hhex*	TGTGGTCTCCGTTCATCCAG	TTTGACCTGTCTCTCGCTGA
*pax8*	GAAGATCGCGGAGTACAAGC	CTGCACTTTAGTGCGGATGA
*tra*	CAATGTACCATTTCGCGTTG	GCTCCTGCTCTGTGTTTTCC
*dio2*	TTCTCCTTGCCTCCTCAGTG	AGCCACCTCCGAACATCTTT
*nkx2.1*	AGGACGGTAAACCGTGTCAG	CACCATGCTGCTCGTGTACT
*tg*	CCAGCCGAAAGGATAGAGTTG	ATGCTGCCGTGGAATAGGA
*tshβ*	GCAGATCCTCACTTCACCTACC	GCACAGGTTTGGAGCATCTCA
*chata*	AGGGAATAGTGCTTGTGCAG	GCTGGAAGTTCACTCATGCT
*ngfb*	CGCCATTGGAACTCATATTG	CACGCAAGCTACATTGATCC
*creb1a*	ATTAGCCAATAACGGGACGG	CCACTACTTGATTGCTGGGAAC
*chrna7a*	CTCCTGGACGTATGGAGGAT	ACTTCCACAAGGTCCCACTC
*nqo1*	CTCAAGGATTTGCCTTCAGC	CGCAGCACTCCATTCTGTAA
*Crhb* (*crf*)	ATCTCAAGGAAGGCGAATAGA	AACATCGATGGAAAGTGATGA
*mao*	CAACAACCTCTGGAGGACA	GTTGCTGCATGGTCATCTT
*mc2r*	TGAGTCACGCTGTTATTGATCC	AGATCCTTGAAGCTGAGGACAG
*pomc*	GAATCCGCCGAAACGCTTCC	GGGTCCTTCTTTCCAAGTGGGTTT
*prl*	GCTCGGTCTCTGCTGTTG	GGTGTTGCGTTCTGGATGT

## Data Availability

The data that support the findings of this study are available from the corresponding author upon reasonable request.
